# Left Ventricular Wall Reconstruction with Autologous Vascularized Tunica Muscularis of Stomach in a Porcine Pilot Model

**DOI:** 10.1159/000522478

**Published:** 2022-02-08

**Authors:** Tobias Schilling, Tanja Meyer, Gudrun Brandes, Dagmar Hartung, Igor Tudorache, Ingo Nolte, Frank Wacker, Andres Hilfiker, Klaus Höffler, Axel Haverich, Serghei Cebotari

**Affiliations:** ^a^Department of Cardiothoracic, Transplantation, and Vascular Surgery, Medical School Hannover, Hannover, Germany; ^b^Institute of Neuroanatomy and Cell Biology, Medical School Hannover, Hannover, Germany; ^c^Institute for Radiology, Hannover Medical School, Hannover, Germany; ^d^Clinic for Small Animals, University of Veterinary Medicine Hannover Foundation, Hannover, Germany

**Keywords:** Angiogenesis, Animal model, Experimental model, Heart, Regeneration, Stomach, Surgery, Vascularization

## Abstract

**Introduction:**

Surgical replacement of dysfunctional cardiac muscle with regenerative tissue is an important option to combat heart failure. But, current available myocardial prostheses like a Dacron or a pericardium patch neither have a regenerative capacity nor do they actively contribute to the heart's pump function. This study aimed to show the feasibility of utilizing a vascularized stomach patch for transmural left ventricular wall reconstruction.

**Methods:**

A left ventricular transmural myocardial defect was reconstructed by performing transdiaphragmatic autologous transplantation of a vascularized stomach segment in six Lewe minipigs. Three further animals received a conventional Dacron patch as a control treatment. The first 3 animals were followed up for 3 months until planned euthanasia, whereas the observation period for the remaining 3 animals was scheduled 6 months following surgery. Functional assessment of the grafts was carried out via cardiac magnetic resonance tomography and angiography. Physiological remodeling was evaluated histologically and immunohistochemically after heart explantation.

**Results:**

Five out of six test animals and all control animals survived the complex surgery and completed the follow-up without clinical complications. One animal died intraoperatively due to excessive bleeding. No animal experienced rupture of the stomach graft. Functional integration of the heterotopically transplanted stomach into the surrounding myocardium was observed. Angiography showed development of connections between the gastric graft vasculature and the coronary system of the host cardiac tissue.

**Conclusions:**

The clinical results and the observed physiological integration of gastric grafts into the cardiac structure demonstrate the feasibility of vascularized stomach tissue as myocardial prosthesis. The physiological remodeling indicates a regenerative potential of the graft. Above all, the connection of the gastric vessels with the coronary system constitutes a rationale for the use of vascularized and, therefore, viable stomach tissue for versatile tissue engineering applications.

## Introduction

Chronic left-sided heart failure is one of the most common cardiovascular diseases in western societies [1]. In severe cases, surgical reconstruction of the ventricle can be performed. This surgical intervention aims for both the reduction of the enlarged ventricle and the restoration of its physiological ellipsoid shape [2]. Synthetic patch materials, such as Dacron, are often required for the reconstruction [3]. However, synthetic materials cannot undergo growth and do not have a regenerative potential. Finally, such akinetic or dyskinetic grafts do not actively contribute to the ventricular output with subsequent disadvantages especially after large scale tissue replacement.

Therefore, biological and regenerative approaches have been attempted [4], such as supporting ischemic myocardium with skeletal muscle [5, 6]. These approaches were driven by the anticipation of physiological remodeling of the applied tissues and their acquisition of specific myocardial functions such as contraction and conduction of cardiac excitation. The substrates need to withstand an intracardiac blood pressure of up to 240 mm Hg when used as patch material for the left ventricle. This mechanical requirement calls for stronger tissues that usually have a thicker diameter. However, supplying oxygen and nutrients via diffusion, as well as removing metabolic wastes, becomes problematic for graft tissues of a thickness greater than 100 μm [7]. Adequate vascularization of the substitute myocardial tissue is a key to ensure viability and functionality. Therefore, we employed a stomach piece, including its arterial and venous vessels and their native connection to the body circuit, which we translocated from the abdomen to the thoracic cavern via the diaphragm. Hence, in the present study, we examined the applicability and the physiological integration of autologous vascularized stomach tissue as a prosthetic material for the full wall replacement of dysfunctional left ventricular myocardium in a swine model.

## Materials and Methods

### Study Design

All experiments were carried out according to the European Convention on Animal Husbandry and the ARRIVE guidelines where applicable [8]. The study was approved by a competent authority (LAVES, Niedersächsisches Landesamt für Verbraucherschutz und Lebensmittelsicherheit, Lower Saxony, Germany) in accordance with Article 8 Paragraph 1 of the Animal Welfare Act, Civil Code 1. IS 01484 (#07/1353, and #08/1604). The Ruthe Teaching and Research Farm of the University of Veterinary Medicine Hannover, Foundation provided all animals.

A left ventricular transmural myocardial defect was covered with an autologous vascularized segment of stomach tissue in Lewe mini pigs (*n* = 6) with an average weight of 31 kg. No pig showed signs of clinical impairments prior to surgery, which would have been an exclusion criterion. Euthanasia and explantation of the grafts were planned 3 (group 3M), and 6 (group 6M) months after surgery. Three animals served as a control group, in which the myocardial defect was covered by a Dacron patch (Dacron group, *n* = 3).

### Surgical Technique

Heart surgery was performed under general anesthesia with endotracheal intubation. Anesthesia was induced with ketamine (20 mg/kg body weight intramuscularly i.m.; B. Braun, Melsungen, Germany) and azaperone (2 mg/kg body weight (i.m.); Janssen, Germany) and was sustained with 2% inhalative isoflurane in oxygen. Analgesia was carried out with the administration of fentanyl (5 μg/kg body weight (i.v.); Janssen-Cilag, Neuss, Germany) 150 mg of amiodarone was administered as anti-arrhythmic prophylaxis.

First, a circular piece of the large curvature of the stomach with a diameter of 4 cm was isolated via median laparotomy while maintaining the gastroepiploic vascular supply. The resulting defect of the stomach was immediately closed with a continuous suture (PDS 2.0; Ethicon, Norderstedt, Germany). Surgical access to the thoracic cavity was achieved via an expansion of the laparotomy as a left-lateral thoracotomy in the fourth intercostal space. The cannulas for cardiopulmonary bypass (Stöckert S3; Sorin Group Germany GmbH, München, Germany) were placed into the carotid artery and via the inferior vena cava into the right atrium. The cardiopulmonary bypass was started following systemic heparinization (400 international units [IU]/kg body weight; Heparin-Natrium-25000; Ratiopharm, Ulm, Germany) and a flow rate of 60–80 mL/kg body weight/min aiming for a mean blood pressure of 50–60 mm Hg at a body temperature of 28–30°C. The body temperature of the animal was cooled to 28°C because deep hypothermia was shown to have a preventive effect against heart rhythm disorders of the test animals in preliminary test series.

**Fig. 1:**
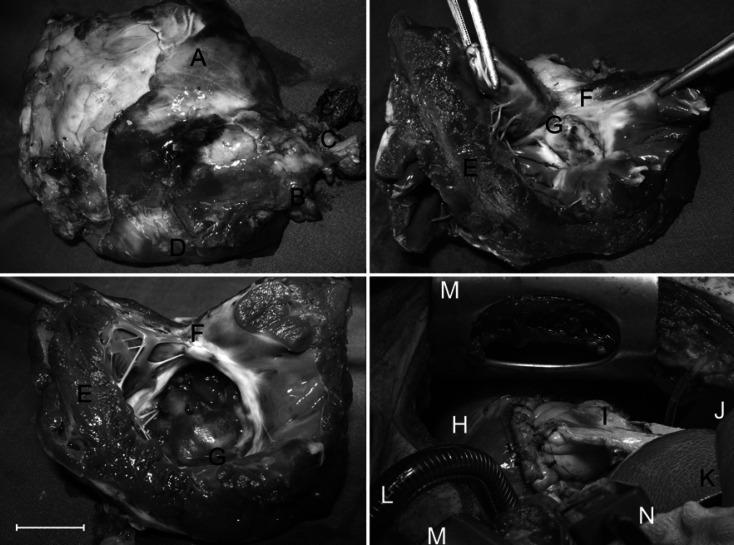
TOP LEFT: Explanted porcine heart following transplantation of a vascularized autologous segment of the stomach. A: Anterolateral face of left ventricle; B: Autologous transplanted segment of the stomach; C: Pedicle with gastroepiploic artery; D: Fibrotic cover of border zone between native myocardium and transplanted stomach. TOP RIGHT: Opened explanted left ventricle 6 months following surgery. E: Native myocardium of left ventricle; F: Solid scar tissue in the border zone of myocardium and transplanted segment of stomach; G: Autologous vascularized segment of contractible stomach with small aneurysmal dilatation. BOTTOM LEFT: Opened explanted left ventricle 6 months following surgery. E: Native myocardium of left ventricle; F: Solid scar tissue in the border zone of myocardium and transplanted segment of stomach; G: Autologous vascularized segment of contractible stomach with small aneurysmal dilatation. BOTTOM RIGHT: intraoperative site after fixation of a segment of the stomach with a diameter of 4 cm to the anterolateral face of the heart. H: Left ventricle. Ramus interventricularis of left coronary artery (RIVA); I: Segment of stomach with pedicle incl. native left gastroepiploic artery and vein; J: Diaphragma; K: Spleen; L: Venous cannula of heart-lung machine for extracorporeal circulation; M: Retractor; N: Blood aspirator of heart-lung machine Bar indicates 2 cm.

After opening the pericardium and following induction of myocardial fibrillation, a piece of myocardium with a diameter of approximately 4 cm was resected from the anterolateral area of the left ventricle. Then, the excised piece of the stomach including the vascular pedicle was transdiaphragmatically transferred into the thoracic cavity. The lamina mucosa was mechanically removed before the patch was grafted into the left ventricular defect using the single-button technique (Polyprolene 4.0; Ethicon, Norderstedt, Germany) (Fig. 1, bottom right). A nonresorbable Dacron patch was used for surgery of the control animals instead of the stomach segment.

The heparin effect was then antagonized by protamine (400 IU/kg body weight; Medapharma, Dübendorf, Switzerland). After de-cannulation hemostasis, reheating to 37°C body temperature and stabilization of circulation was carried out. Then, the readaptation of the ribs (Mersilene 2.0; Ethicon, Norderstedt, Germany) as well as the muscle layers of both the thoracotomy and laparotomy (Vicryl 2.0; Ethicon, Norderstedt, Germany) was performed using continuous suture. This was followed by a Donati suture (CBX1 Vicryl; Ethicon, Germany) and sealing of the wound with aluminum spray (Almapharm, Germany).

### Cardiac MRI

Cardiac magnetic resonance imaging (MRI) was performed with a 1.5 Tesla MR scanner (Genesis Sigma CVI; GE Healthcare, Solingen, Germany). The MRI investigation of the animals took place under general anesthesia 3 months following surgery and immediately prior to scheduled euthanasia. Before the MRI examination, anesthesia of the animals was induced with ketamin (20 mg/kg body weight i.m., B. Braun) and propofol (propofol-lipuro 1%, 4–6 mg/kg body weight, i. v.; B. Braun, Melsungen, Germany) and maintained after that with inhalative 2% isoflurane in oxygen.

Cardiac MRI was done using a four-element phased array receiver coil. ECG gated, breath-hold balanced steady-state free precession gradient echo sequence (FIESTA) in the short-axis view were used for the quantitative evaluation of left ventricular volumes and function. Additionally, late enhancement imaging was performed using a T1-weighted inversion recovery gradient echo of a series of short axes. For quantitative analysis, endocardial and epicardial contours were traced manually in all end-systolic and end-diastolic short-axis slices between the atrioventricular plane and the apex of the heart using cvi42 software version 4.1 (Circle Cardiovascular Imaging Inc., Calgary, AB, Canada).

### Angiography

Angiography was carried out immediately prior to euthanasia via injection of phenobarbiturate (450 mg/kg body weight; WDT, Wertingen, Germany) under general anesthesia. A median sterno-laparotomy was chosen as the surgical access path. After systemic heparinization (400 IU/kg body weight; Heparin-Natrium-25000; Ratiopharm), the graft's gastroepiploic artery was incised to allow the antegrade insertion of a cannula (Vasofix® Safety, 22G; B. Braun, Melsungen, Germany). A nonionic contrast agent (Imeron 350®; Bracco-Byk Gulden, Konstanz, Germany) was applied via the cannula. The screening was performed using a C-arm *X*-ray unit.

### Histology

The explanted graft tissue was stained with hematoxylin-eosin and Movat pentachrome for histological characterization. For the Movat pentachrome staining, the samples were fixed in 3.5% formaldehyde solution (Otto Fischer GmbH & Co. KG, Saarbrücken, Germany), dehydrated in alcohol solutions of increasing concentration, and embedded in paraffin (Carl Roth GmbH & Co. KG, Karlsruhe, Germany). Subsequently, 10-µm-thick sections were stained with Movat pentachrome (Merck KGaA, Darmstadt, Germany and Waldeck GmbH & Co. KG, Münster, Germany). The samples of the control group were embedded in Technovit 9100 (Heraeus, Hanau, Germany).

For the immunohistochemical analysis, samples were fixed in Tissue Tek (Sakura Finetek, Torrance, CA, USA) and flash frozen with liquid nitrogen (Messer Griesheim GmbH, Krefeld, Germany). To differentiate between the smooth musculature of the stomach tissue and the myocardium, double staining with different antigen specificity for troponin T and the myosin heavy chain proteins of the smooth muscle cells was performed. Connexin 43 was used to reveal gap junctions. The general cell nucleus staining was performed using 4′,6-diamidine-2-phenylindole.

## Results

### Clinical Results

Eight out of nine animals survived the surgical procedure without significant postoperative complications until planned termination of the observation period. One animal died intraoperatively because of excessive bleeding due to unmanageable leakage of the anastomosis. No animal died postoperatively due to rupture of the gastric graft. Several days after the procedure, the animals exhibited normal eating behavior and activity. Neurological abnormalities were not observed. No wound infections occurred.

### Magnetic Resonance Imaging

**Fig. 2:**
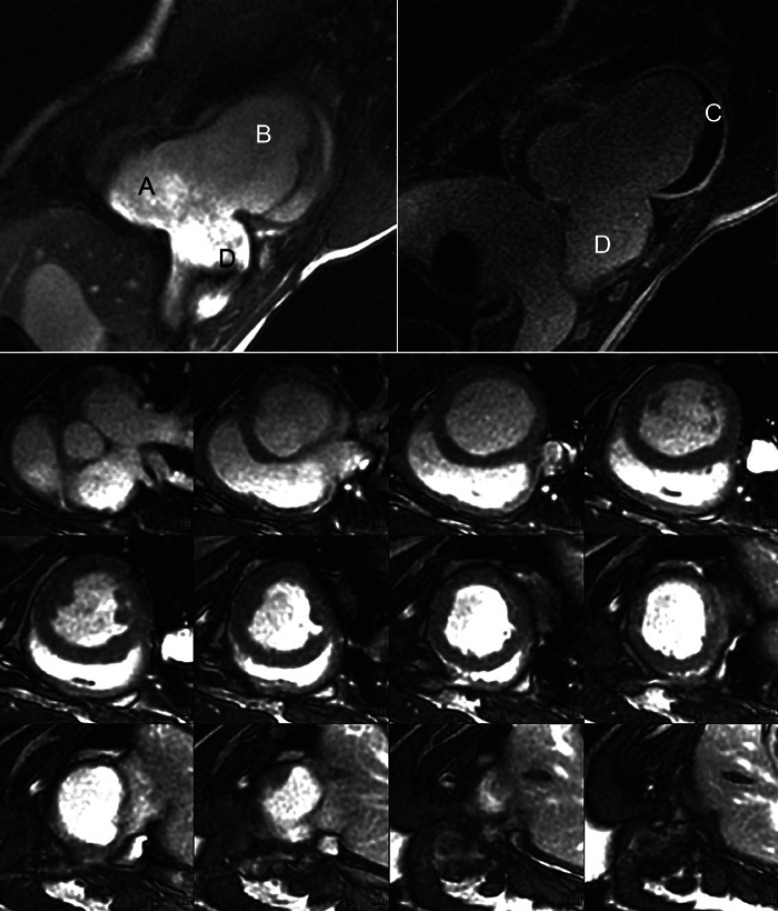
TOP: Cardiac MRI of pig (Group A) following 3 months after reconstruction of a transmural left ventricular lesion with a diameter of 4 cm. TOP LEFT: SSFP gradient echo sequence (FIESTA) in standard short-axis view. A: Left ventricle; B: Large aneurysm; C: Thrombus; D: Covered perforation. TOP RIGHT: Late enhancement in standard short-axis view. C: Thrombus; D: Covered perforation. BOTTOM: Cardiac MRI SSFP gradient echo sequence (FIESTA) in standard short-axis view of pig (group 6M) following 6 months after surgery from base to apex of the left ventricle. SSFP, steady-state free precession.

The animals of the Dacron group reached a mean LVEF of 71.3%. Overall, the left ventricular ejection fractions of the animals in the sample ranged from 10 to 55%. The MRI examination revealed a covered perforation of the graft in the animal with the intraventricular thrombus (Fig. 2, top C, D). There was dilatation with hypokinesia of the left ventricular myocardium in the boundary between the myocardium and the stomach tissue in other animals but no rupture of the gastric patch (Fig. 2, top B, bottom).

An improvement of the pumping function of the left ventricle in one animal took place over the course of the observation period. There was even a regression of a small aneurysm formation in one animal of group 6M (6 months).

A late enhancement was consistently detected in the boundary zone between the myocardium and the stomach patch. This indicates the formation of connective tissue as part of the biological integration of the graft into the myocardium (Fig. 2, top).

### Angiography

**Fig. 3:**
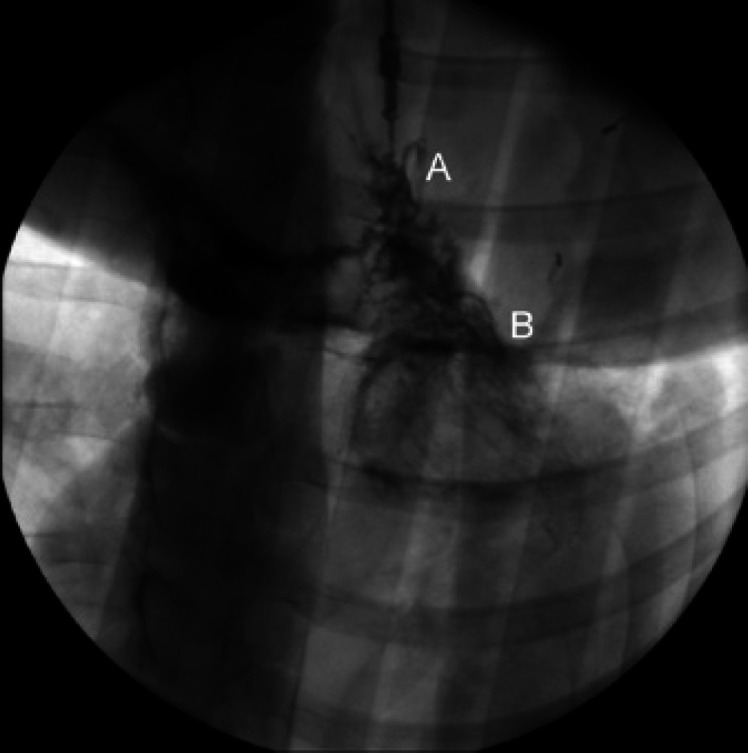
Angiography of gastroepiploic artery 3 months following reconstruction of the left ventricular lesion using an autologous vascularized segment of the stomach. A: Pedicle of the segment of stomach with contrast agent applied into gastroepiploic artery; B: Lateroanteral face of left ventricle with retrograde filling of coronary vessels with contrast agent.

No stenoses, embolisms, or aneurysms of the grafts' vasculature were diagnosed. There was no evidence of insufficiently perfused regions of the grafts. The antegrade infusion of contrast agent into the gastroepiploic artery flowed into the native coronary vessels of the myocardium in each animal (Fig. 3). The movements of the stomach segment indicated beat-synchronous motility.

### Macroscopic Findings

There were connective tissue adhesions between the transplanted stomach segment, the surrounding epicardium, the pericardium, and the pleura (Fig. 1, top left). After opening the explanted hearts, there was the pleated aspect of the contractile muscular stomach wall visible (Fig. 1, top right and bottom left G). The boundary zone between the myocardium and stomach presented as solid white scar tissue with striated offshoots into the surrounding endocardium (Fig. 1, top left D; top right F, bottom left F).

The operative site during explantation revealed a dilatation of the transplanted stomach. The surrounding thoracic organs showed no pathological changes. In one animal (group 6M), there was a thrombus alongside an aneurysm.

### Histology and Immunohistochemistry

The explanted tissues of the control group revealed a foreign body reaction to the synthetic fabric. Fiber-rich connective tissue with high capillary density formed between the Dacron patches and the myocardium (online suppl. Fig. 1; for all online suppl. material, see www.karger.com/doi/10.1159/000522478). A cell-rich neointima covered the Dacron tissue.

**Fig. 4:**
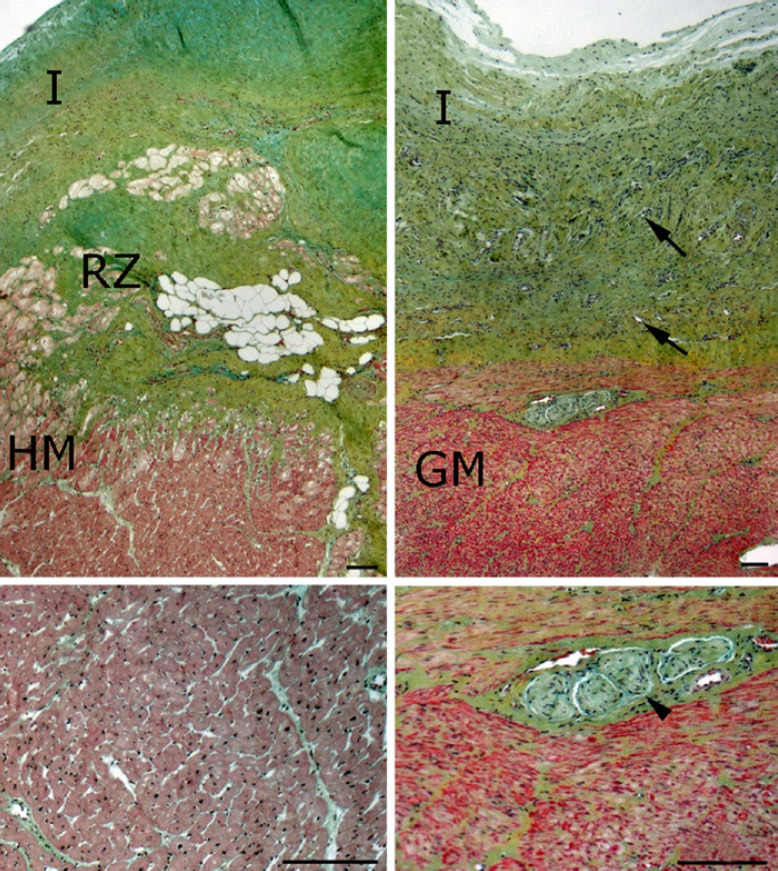
Border zone between myocardium (HM) and tunica muscularis of gastric tissue (GM). A continuous initima (I) covers the myocardium (HM) as well as the gastric patch (GM). Cells of the conductive systems (RZ) are present. Triangle indicates myenteric ganglion. Pentachrome staining. Bars indicate 100 µm.

In the experimental groups there was integration of the various tissues. The conduction system presented as Purkinje fibers, and there were a large number of capillaries in all explants (Fig. 4).

The boundary zone between the myocardium and stomach tissue was characterized by granulation and scar tissue. Thick collagen fiber bundles were adjacent to vascularized granulation tissue with macrophages and stromal cells. Neutrophilic granulocytes were visible, but not abundant in all explants. Myofibroblasts were found in the gastric patch (Fig. 4).

The transplanted stomach tissue consisted of the tunica muscularis and tunica serosa. Spindle-shaped smooth muscle cells were found in the inner and outer ring layers. Neurons of the myenteric plexus were detected (Fig. 4).

There was a continuous single-layer of endothelial cells forming a neointima on the transplanted stomach tissue (Fig. 4). There were no signs of necrosis, degeneration, or infection.

Using double staining against cardiac troponin and the myosin of the smooth muscle cells, both muscle types were detected in all explanted samples. Stained blood vessels were ubiquitously detectable (online suppl. Fig. 2, top). Immunohistochemical staining with connexin 43 revealed the connection between cardiomyocytes and cells of the grafts (online suppl. Fig. 2, bottom).

## Discussion

We introduced a procedure for a transmural left ventricular reconstruction of dysfunctional myocardium with a vascularized graft. Overall, this two-cavity intervention in the pig model represents a considerable challenge to the surgical, anesthesiological, veterinary, and technical requirements. Nevertheless, our results suggest good biological integration of the graft into the hosts' myocardium, the possibility of functional improvement, and most notably connection of the gastric vasculature to the coronary system.

### Regenerative Capacity via Physiological Remodeling

Reconstructing dysfunctional heart muscle with regenerative materials is important because of the increasing incidence and prevalence of severe heart failure [9]. Several biological substrates have been assessed as regenerative patch materials [10, 11, 12, 13, 14, 15]. The major motivation for the use of viable biological tissue is its potential for physiological remodeling. The connection to the electrical cardiac conduction system via gap junctions is also essential in order to synchronize the contraction phases of both the heart and graft. In our study, we observed good integration of the stomach tissue into the left ventricular myocardium. Only one animal with the series' largest aneurysmal formation of the graft showed a thrombus in the lumen of the transplanted gastric tissue. Blood that stagnated in this pronounced aneurysm is likely the cause for the thrombus.

We were able to observe the pulse-synchronized movement of stomach tissue in the MRI and upon explantation of the heart. It remains unclear whether the smooth muscle cells of the tunica muscularis of the stomach took over a rhythmic contractile function. Nevertheless, Ota et al. [16] were also able to measure moderate electrical activity from porcine urinary bladder after it was used to cover defects in the right ventricle in pigs.

### Mechanical Stability and Function

Most biological tissue grafts are unable to withstand the high pressure loads present in the left ventricle. Thus, most groups employ biological grafts only for reconstructive surgery of the right ventricle or atrium [17]. In the current study, the feasibility of covering a transmural left ventricular myocardial defect with a piece of autologous vascularized stomach was demonstrated in our swine model. Despite the dilation of the graft tissue in one animal, 5 out of 6 test animals showed no rupture of the transplanted stomach tissue. Maybe an iatrogenic damaging of the stomach's tunica muscularis while removing the tunica mucosa has caused the covered perforation in the one animal with the large aneurysm. Moreover, an improved left ventricular ejection fraction over time could be detected in one animal, indicating the potential regenerative capacity of the graft.

### Vascularization

A common problem of the previously tested approaches to using biological myocardial prostheses is the lack of vascularization [4]. Only tissue with a thickness of up to 100 μm can be adequately supplied by diffusion [7]. Only pre-vascularized tissue would warrant supply with oxygen, nutrition, and evacuation of products of metabolism and simultaneously sufficient stability. In our current work, the connection of gastric vascularized tissue to the arterial and venous supply of the myocardium was demonstrated.

Ruel et al. [18] transplanted an autologous segment of the stomach transdiaphragmatically onto the ischemic myocardium and observed improved perfusion in this area. But, Ruel et al. [18] fixed the stomach tissue to the myocardium just epicardially. In our study, we were able to demonstrate the functional integration of the vascular supply of the transplanted stomach tissue by a complete transmural left ventricular wall replacement.

### Limitations

The contribution of the graft's smooth muscle cells and its physiological remodeling to the heart's pump function cannot be distinguished by the results of this study. In order to statistically prove the observed positive effect, a higher number of tests would be required. Finally, the presumed long-term superiority of regenerative myocardial prostheses over conventional methods should be evaluated in controlled comparative trials.

## Conclusions

The results of this study provide evidence that an improvement in heart function through full left ventricular wall reconstruction with vascularized grafts may be feasible, yet technically demanding. However, the observed dilation of some grafts indicates that stomach tissue does not initially have the required full mechanical stability to withstand the high pressure of the left ventricle in all cases. In order to use stomach tissue as left ventricular myocardial prostheses, additional mechanical stabilization would foster the safety of this therapeutic approach.

Nevertheless, the improvement of cardiac function, the good physiological integration, as well as the connection of the gastric vessels with the coronary system of the hosts verify the potential of stomach tissue as a regenerative myocardial prosthesis. Finally, the use of vascularized tissue as demonstrated in this study would facilitate further tissue engineering concepts for many surgical fields, which − so far − are not clinically applicable because of the lack of sufficient vascular supply.

## Statement of Ethics

All experiments were carried out under the European Convention on Animal Husbandry and approved by a competent authority (LAVES, Lower Saxony) in accordance with § 8 Paragraph 1 of the Animal Welfare Act, Civil Code 1. IS 01484. The decision reference numbers of the LAVES are #07/1353 and #08/1604.

## Conflict of Interest Statement

The authors have no conflicts of interest to declare.

## Funding Sources

The authors are grateful to the German Research Foundation (SFB599, project R7) for funding this project.

## Author Contributions

Our multidisciplinary group of physicians, veterinarians, biologists, and technicians carried out the work of the manuscript at hand. Tobias Schilling, MD and Axel Haverich were responsible for the concept development and discussion of the experiments' findings. Additionally, Tobias executed the project management, surgical procedures, investigation of the explants, and MRI- and angiography results, as well as the writing of this manuscript. As veterinarians, Tanja Meyer and Ingo Nolte took care of the development of the specific veterinary perioperative animal care protocols and the pre-, peri, and postoperative care of the animals. They also monitored the clinical status of the animals. Tanja contributed to the preparation of the explants for further analysis and the writing of this manuscript. Gudrun Brandes, MD, prepared the explants for light microscopy and electron microscopy and carried out their microscopic characterization. Andres Hilfiker supported this characterization with his expertise as a biologist. Dagmar Hartung, MD, and Frank Wacker, MD, adapted established MRI procedures to the specific veterinary frame of the conducted experiments, which they eventually executed and analyzed. Igor Tudorache, MD, and Serghei Cebotari, MD, developed the surgical procedures, contributed to the surgery and the angiographic investigations. Klaus Höffler is a perfusionist who developed and adjusted extracorporeal circulation protocols to the specifics of the challenging animal model described in this manuscript. He also evaluated and monitored the adequate connection technique, the hemodynamic regime, and the chemical parameters.

## Data Availability Statement

The datasets used and/or analyzed during the current study are available from the corresponding author on reasonable request.

## Supplementary Material

Supplementary data

Supplementary data

Supplementary data

## Notes

Tobias Schilling and Tanja Meyer contributed equally to this work.
