# Supplementary material for: Left Ventricular Wall Reconstruction with Autologous Vascularized Tunica Muscularis of Stomach in a Porcine Pilot Model
Source: Eur Surg Res. 2022 Feb 8;63(4):219–26. doi: 10.1159/000522478 (PMC9808675; doi:10.1159/000522478)
Supplement: Supplementary file 1 — Supplementary data [file esr-0063-0219-s01.docx]

**Supplement fig. 1.** Border zone between myocardium (HM) and Dacron (D). Pentachrome staining. Arrows indicate cardiomyocytes. Triangles indicate giant cells within cell rich granulation tissue surrounding Dacron filaments. Bars indicate 100µm.

**Supplement Fig. 2.** TOP: Double staining of cardiac troponin (red) and smooth muscle cell myosin (green). Ubiquitous vascular structures. Immunohistochemical staining. Cellular nuclei were stained with DAPI (blue). Bar indicates 2mm

BOTTOM: Vascularized (V) border zone between myocardium and gastric patch. Immunohistochemical staining of Connexin 43. Bar indicates 200µm
