# Supplementary figures and images for: Left Ventricular Wall Reconstruction with Autologous Vascularized Tunica Muscularis of Stomach in a Porcine Pilot Model
Source: Eur Surg Res. 2022 Feb 8;63(4):219–26. doi: 10.1159/000522478 (PMC9808675; doi:10.1159/000522478)

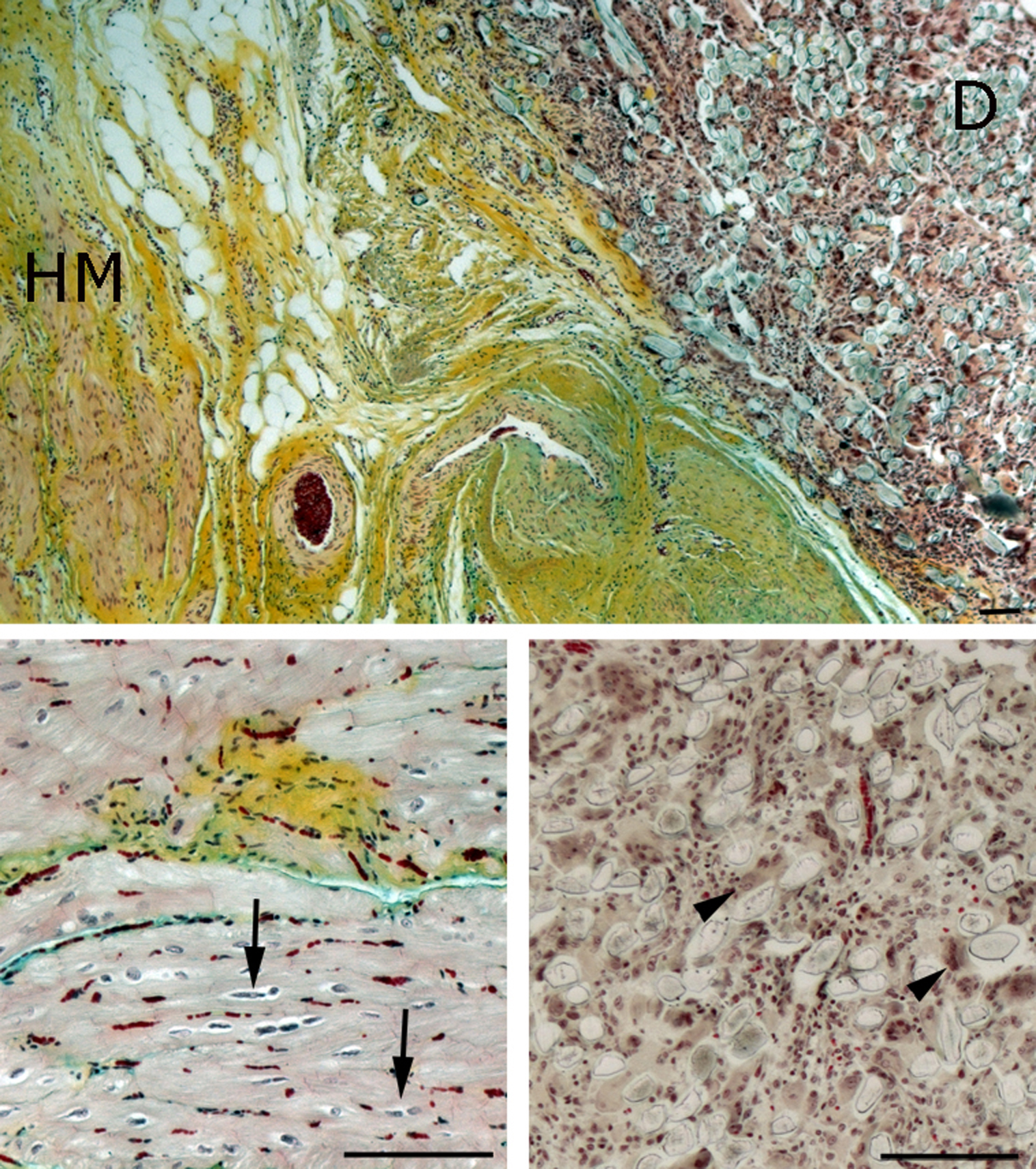

Supplement: Supplementary file 2 — Supplementary data [file esr-0063-0219-s02.jpg]

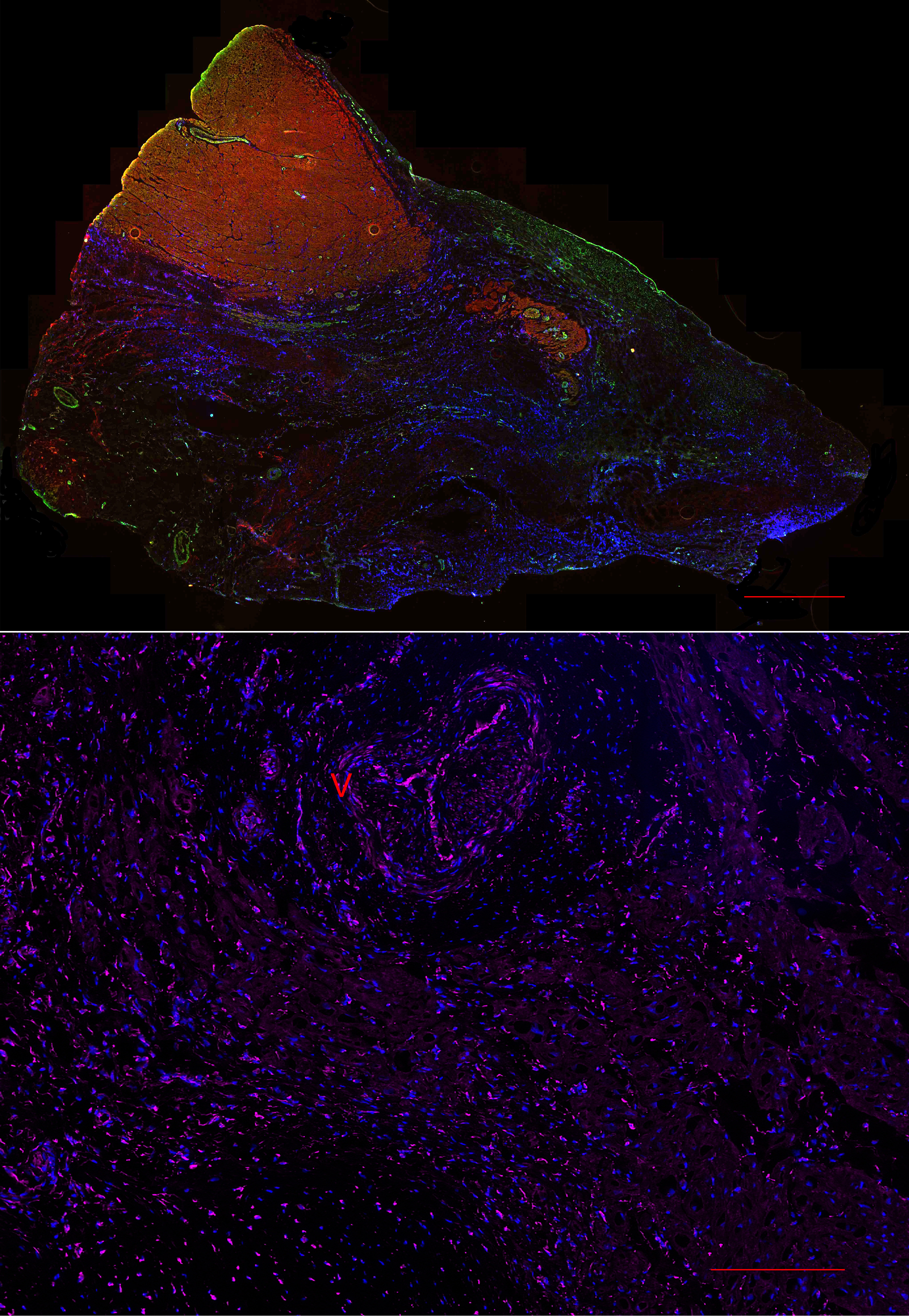

Supplement: Supplementary file 3 — Supplementary data [file esr-0063-0219-s03.jpg]
